# AmericaPlex26: A SNaPshot Multiplex System for Genotyping the Main Human Mitochondrial Founder Lineages of the Americas

**DOI:** 10.1371/journal.pone.0093292

**Published:** 2014-03-26

**Authors:** Alexandra Coutinho, Guido Valverde, Lars Fehren-Schmitz, Alan Cooper, Maria Inés Barreto Romero, Isabel Flores Espinoza, Bastien Llamas, Wolfgang Haak

**Affiliations:** 1 Australian Centre for Ancient DNA, School of Earth & Environmental Sciences, The University of Adelaide, Adelaide, South Australia, Australia; 2 Historical Anthropology and Human Ecology, Johann-Friedrich-Blumenbach Department of Zoology and Anthropology, University Goettingen, Goettingen, Germany; 3 Department of Anthropology, University of California Santa Cruz, Santa Cruz, California, United States of America; 4 Museo de Sitio Huaca Pucllana, Miraflores, Lima, Perú; University of Perugia, Italy

## Abstract

Phylogeographic studies have described a reduced genetic diversity in Native American populations, indicative of one or more bottleneck events during the peopling and prehistory of the Americas. Classical sequencing approaches targeting the mitochondrial diversity have reported the presence of five major haplogroups, namely A, B, C, D and X, whereas the advent of complete mitochondrial genome sequencing has recently refined the number of founder lineages within the given diversity to 15 sub-haplogroups. We developed and optimized a SNaPshot assay to study the mitochondrial diversity in pre-Columbian Native American populations by simultaneous typing of 26 single nucleotide polymorphisms (SNPs) characterising Native American sub-haplogroups. Our assay proved to be highly sensitive with respect to starting concentrations of target DNA and could be applied successfully to a range of ancient human skeletal material from South America from various time periods. The AmericaPlex26 is a powerful assay with enhanced phylogenetic resolution that allows time- and cost-efficient mitochondrial DNA sub-typing from valuable ancient specimens. It can be applied in addition or alternative to standard sequencing of the D-loop region in forensics, ancestry testing, and population studies, or where full-resolution mitochondrial genome sequencing is not feasible.

## Introduction

Population genetic studies on modern-day Native American populations have described the presence of five haplogroups (hgs), termed A, B, C, D and X [1]–[3]. These five hgs are shared with East Asian populations and support an entry route to the Americas via the Bering landmass. However, Native American populations can be distinguished from their East Siberian source populations by exhibiting distinct sub-haplogroups (sub-hgs), which can only be found in the Americas. These so-called ‘founder lineages’ have been used to describe the demographic history of Native American populations and to shed light on the timing of the entry into and spread throughout the Americas [4]–[6]. The fact that the mtDNAs of all human populations native to the Americas can be assigned to one of the founder lineages pertains to stochastic events that would have affected the initial colonizers of the Americas [7]. The low genetic variation found in modern Native American groups is believed to be due to either population bottlenecks or genetic drift [8], [9].

Most mitochondrial DNA (mtDNA) studies on prehistoric American populations involve sequencing of the D-loop, which contains Hypervariable Regions 1 and 2 (HVR1 and HVR 2 respectively), to describe a sequence haplotype, from which the hg can be inferred [10], [11]. Sequencing of the HVR regions of mtDNA was relatively cost-effective and less time consuming than full mtDNA sequencing, and is therefore still the method of choice for many labs which study human populations [11]. Yet not all lineages harbour enough variation in the D-loop from which to infer a sub-hgs at a deeper level than the overall hg, let alone a specific founder lineage [11]–[14]. As a result, many past and present studies on Native American population history have been restricted to the information gained from the distribution of the major five Pan-American hgs.

The coupling of multiplex polymerase chain reaction (PCR) with a Single Base Extension (SBE) reactions, based on the established SNaPshot (Applied Biosystems) or minisequencing principle, has been widely used to design panels of single nucleotide polymorphisms (SNPs) for forensic and anthropological studies [15]. It has also found wide use in population genetic studies focussing on mtDNA and Y-chromosome SNPs, either including SNPs with a global representation or via a targeted selection of characteristic SNPs representing specific geographic regions [13], [16]–[21]. The design of a SNP panel including those markers defining the 15 American founder lineages described by Perego et al. [22] and more had not been attempted, although ‘Multiplex 3’ in van Oven et al. 2011 [23] covered 12 out of these 15. The primary aim of this study was therefore to design a novel SNaPshot assay that enables a fast and cost-efficient high-resolution typing of the majority of known Native American sub-hgs by targeting 26 characteristic SNPs. Our goal was to develop an assay that is universally applicable to accommodate the specific needs of damaged and degraded DNA in ancient DNA work and forensics. Selective sequencing of the SNP regions of interest not only allows for flexibility in the number and choice of SNP sites but also allows (with reservations) the design of ultra-short amplicon lengths (50–80 bp) suitable for degraded DNA typing [13], while using far less DNA than traditional sequencing methods or SNP-typing in individual singleplex PCRs. This is of great importance in forensic and ancient DNA studies where sample DNA is a limited resource [23], [24]. The secondary aim was to develop an assay that could complement an established assay with a global set of SNPs (GenoCore22, see [24] but also [23]) and at the same time provide a fast and efficient screening tool that allows the assessment of overall sample quality (presence of very short fragments of endogenous mtDNA and absence of contaminant hgs) for further use in mitochondrial genome sequencing via DNA library preparation and targeting enrichment techniques, e.g. [25]–[27].

## Materials and Methods

### AmericaPlex26 SNP selection

We developed a multiplex SNaPshot reaction targeting 26 SNP sites in total including characteristic SNPs of the four major Pan-American sub-hgs A2, B2, C1 and D1, as well as SNP sites for the minor Pan-American lineages C4c, D2a, D4h3a and X2a [6]. The initial choice of SNPs was based on a study by Perego et al. [22] describing 15 American founder lineages. Additional SNP sites were chosen for sub-hgs within each major hg based on the most up-to-date mtDNA phylogeny available at the time (phylotree.org, mtDNA tree Build 13, 28 Dec 2011) in order to enhance the discriminating power of the assay. For sub-hgs defined by more than one characteristic SNP we employed selection criteria during the primer design stage based on the ability to design primers with high specificity in the short flanking region around the SNPs, and under a consensus-melting temperature for all pairs in a multiplex environment.

**Figure 1:**
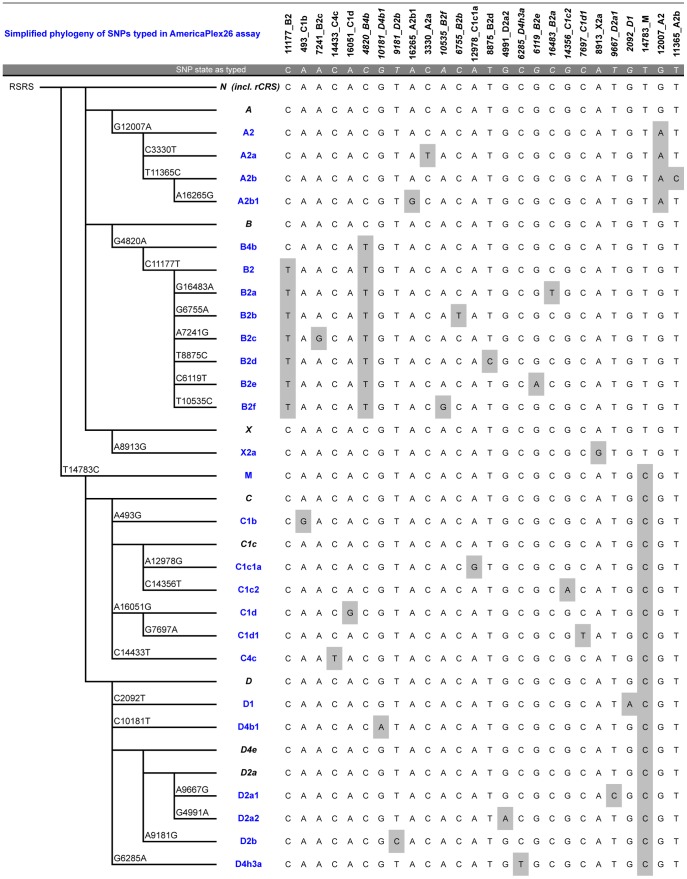
Simplified phylogeny in related to the Reconstructed Sapiens Reference Sequence (RSRS [52]) and typing scheme of the 26 SNPs targeted in the AmericaPlex26. Sub-haplogroups, which can be unambiguously assigned, are shown in blue (blue). Basal hgs, which cannot be unambiguously assigned, are also shown (black) in order to illustrate the phylogenetic relationship, but also the inherent limitations of our assay. The phylogenetic position of the revised Cambridge Reference Sequence (rCRS [53]) is indicated within macro-hg N. SNPs with a SBE primer in reverse direction targeting the opposite strand are given in Italics.

Presented below is a summary of each major Native American sub-hg, their distribution throughout the Americas, as well as the SNP sites chosen to represent the hg and their respective sub-hgs. The representative SNPs typed in the AmericaPlex26 are given in parentheses and a simplified tree illustrating the phylogenetic relationship is shown in Figure 1.

**Haplogroup A2** (G12007A) is found throughout the Americas, but its derivatives A2a (C3330T) and A2b (T11365C) are mainly found in the Northern parts of North America in Inuit, Na-Dené and Siberian populations such as Koryaks and Chukchi [22], [28]–[30], whereas particular subgroups of A2a were also reported from Athapaskan territories in the Southwest [30]. A16265G (defining A2b in [22]) was also added to the assay as it was further resolved to represent sub-hg A2b1, which can be found in Eskimoan-speaking populations (such as the Inuit and Yupik) across the Arctic [31].

**Haplogroup B2** (C11177T) is widely distributed throughout the Americas and is nested within hg B4b (G4820A), which has a largely Asian distribution. Studies on modern-day populations describe sub-hgs within B2, which are prevalent in specific geographic areas within the American continents and thus phylogeographically informative [32]. We included diagnostic SNPs for sub-hgs B2a (G16483A, North/Central), B2b (G6755A, ubiquitous), B2c (A7241G, predominantly Central), B2d (T8875C, Central/South), B2e (C6119T, South) and B2f (T10535C, North/Central) to monitor this phylogeographic pattern in ancient Native American populations through time.

**Haplogroup C** is represented in the Americas by sub-hgs C1b, C1c and C1d [22], [32], whereas sub-hgs C1a and C1e are Siberian/East Asian and European sister-clades, respectively. Sub-hg C1b (A493G) can be found throughout South America. Sub-hg C1c is most frequent in Mexico [22] and was split into sub-hgs C1c1a (A12978G) and C1c2 (C14356T), since the immediate flanking region of the two C1c SNPs defining (G1888A and G15930A) were not suitable for primer design. A recent study by Perego et al. [22] has further resolved Central American sub-hg C1d (A16051G), which now includes sub-hg C1d1 (G7697A). Minor Pan-American hg C4c (C14433T) was recently discovered in an ancient sample from British Columbia, and was found to be one of the founding lineages of the Americas based on coalescent age estimates [33], [34].

**Haplogroup D** is found in the Americas as four distinct sub-hgs D1 (C2092T), D2a, D3 and D4h3a [6], [35]. Sub-hg D2a has been found in Sireniki, Yuit, Aleut and Chukchi populations in Siberia and Aleuts from the Commander Islands [31], [36], [37], and has been further resolved into sub-hgs D2a1 (A9667G; including the Saqqaq Paleo-Eskimo [31]), D2a2 (G4991A) and D2b (A9181G) [22]. Recent studies have further revealed sub-hg D3 to be more derived than previously thought and nested within the larger branch D4b1 (C10181T), which currently encompasses three sub-hgs within North American and Siberian native populations: D4b1a, D4b1b′d, and D4b1c [31]. Therefore, we chose the ancestral SNP defining hg D4b1 for our panel (See mtDNA tree Build 13, 2011). In addition, sub-hg D4h3a (G6285A) is distributed along the Pacific coast of the American continents [6] but most frequent in South America.

**Haplogroup X2a** (A8913G) has only been found in a limited number of samples in North American populations as compared to those of A2, B2, C1 and D1, and is therefore described as minor founding lineage in this paper [8], [35].

Lastly, SNP site T14783C was included as control to define macro-hg M, which encompasses hgs C and D. In contrast, this SNP retains the ancestral state in hgs A, B and X, which belong to macro-hg N.

### Primer and probe design

PCR and SBE primers were designed and quality-controlled using default settings and features in the software package Geneious v5.2 (Geneious version (5.2) created by Biomatters. Available from http://www.geneious.com/) and Batchprimer3 v1.0 [38], both based on the program primer3 [39], generally following the guidelines set out in Sanchez et al. 2006 [40]. Amplicon sizes were deliberately kept smaller than 90 bp in length to allow amplification of highly fragmented DNA as typical in forensics and ancient DNA studies. Given how short the flanking regions of each SNP were, which already constrained our selection of suitable SNPs, we could not consider potential polymorphic sites in these areas nor nuclear insertions, and relied on empirical testing of PCR primer efficiency. SBE primers were then ranked according to quality score and orientation (forward or reverse) for efficient use of fluorescent dyes and fragment length spacing. The latter was adjusted to 4 bp by adding poly-CT tails to the 5′end of each SBE primer (Table 1) [41].

**Table 1:** Table showing the details of multiplex primers and SBE probes used in the AmericaPlex26 assay.

		PCR Amplification				Single-Base Extension			
Site	hg	Primer	Primer Sequences (5′ to 3′)	Conc (μM)	length (bp)	SBE Probe Sequences (5′ to 3′)	Conc (μM)	nt	Alleles (Dyes) as detected
**12007**	A2	L11984	CTAGTCACAGCCCTATACTCCCTCT	0.025	73	(ct)^26^cCCTCTACATATTTACCACAACACAATG	0.015	80	G (blue), A (green)
		H12009	TGTTAATGTGGTGGGTGAGTGAGC	0.025					
**3330**	A2a	L03326	ACATACCCATGGCCAACCTCCT	0.022	60	(ct)^14^cCCATGGCCAACCTCCTACT	0.015	48	C (yellow), T (red)
		H03339	GGAATGCCATTGCGATTAGAATGGGT	0.022					
**11365**	A2b	L11359	GCCAACAACTTAATATGACTAGCTTACACA	0.033	80	(ct)^25^CAACTTAATATGACTAGCTTACACAATAGC	0.020	80	T (red), C (yellow)
		H11381	GGGAGTCATAAGTGGAGTCCGTAAAGAGG	0.033					
**16265**	A2b1	L16262	ACTGCAACTCCAAAGCCACCCC	0.011	56	(ct)^15^ACTCCAAAGCCACCCCTC	0.015	48	A (green), G (blue)
		H16272	GGGTGGGTAGGTTTGTTGGTATCCT	0.011					
**11177**	B2	L11163	CCCACCTTGGCTATCATCACCCG	0.025	63	(ct)^7^cCCCGATGAGGCAACCAG	0.020	32	T (red), C (yellow)
		H11182	GTATGTGCCTGCGTTCAGGCGT	0.025					
**16483**	B2a	L16471	GCTCCGGGCCCATAACACTTGG	0.030	70	(ct)^21^ACCAGATGTCGGATACAGTTCA	0.020	64	C (yellow), T (red)
		H16494	ACCCTGAAGTAGGAACCAGATGTCGG	0.030					
**6755**	B2b	L06750	GTCTGAGCTATGATATCAATTGGCTTCC	0.032	90	*(ct)^16^TGGTGTGCTCACACGATAAA*	0.015	52	C (yellow), T (red)
		H06789	TGCTCGTGTGTCTACGTCTATTCC	0.032					
**7241**	B2c	L07224	TCCGGAATGCCCCGACGTTACT	0.023	68	(ct)^9^cTCGGACTACCCCGATGC	0.015	36	A (green), G (blue)
		H07243	ACAGATGATAGGATGTTTCATGTGGTGT	0.023					
**8875**	B2d	L08864	TCCCCTTATGAGCGGGCACAGT	0.029	76	(ct)^18^CGGGCACAGTGATTATAGGC	0.015	56	T (red), C (yellow)
		H08896	TGTGGTAAGAAGTGGGCTAGGGC	0.029					
**6119**	B2e	L06094	TCGTCACAGCCCATGCATTTGT	0.031	76	*(ct)^22^CCAAAGCCTCCGATTATGAT*	0.015	64	G (blue), A (green)
		H06133	AGTCAGTTGCCAAAGCCTCCGA	0.031					
**10535**	B2f	L10528	AGCATTTACCATCTCACTTCTAGGAATACT	0.031	62	*(ct)^16^GAGGATATGAGGTGTGAGCG*	0.020	52	A (green), G (blue)
		H10537	GTAGGGAGGATATGAGGTGTGAGC	0.031					
**4820**	B4b	L04816	GCCCCCTTTCACTTCTGAGTCCC	0.033	56	*(ct)^11^AGGGGTGCCTTGGGTAAC*	0.020	40	C (yellow), T (red)
		H04828	CCGGATGTCAGAGGGGTGCCTT	0.033					
**8913**	X2a	L08905	CGCTCTAAGATTAAAAATGCCCTAGCCC	0.003	59	(ct)^26^AATGCCCTAGCCCACTTCTT	0.015	72	A (green), G (blue)
		H08915	AGGGGTGTAGGTGTGCCTTGTG	0.003					
**14783**	M	L14774	ACCCCAATACGCAAAACTAACCCCC	0.031	76	(ct)^26^CGCAAAACTAACCCCCTAATAAAA	0.020	76	T (red), C (yellow)
		H14804	TGTTGGATGGGGTGGGGAGGTC	0.031					
**493**	C1b	L00474	TTTCCCCTCCCACTCCCATACT	0.033	73	ctcACTCCCATACTACTAATCTCATCAATACA	0.025	32	A (green), G (blue)
		H00511	TAGCAGCGGTGTGTGTGTGCTG	0.033					
**12978**	C1c1a	L12972	ACGCTAATCCAAGCCTCACCCCA	0.018	65	(ct)^18^CCAAGCCTCACCCCACTACT	0.015	56	A (green), G (blue)
		H12993	TTGGGCTGATTTGCCTGCTGCT	0.018					
**14356**	C1c2	L14348	ACCACAACCACCACCCCATCAT	0.015	67	*(ct)^24^GTAGGATTGGTGCTGTGGGT*	0.015	68	G (blue), A (green)
		H14371	TGGGGTTAGCGATGGAGGTAGGA	0.015					
**16051**	C1d	L16049	TCTTTCATGGGGAAGCAGATTTGGG	0.019	62	(ct)^11^GGGGAAGCAGATTTGGGT	0.015	40	A (green), G (blue)
		H16065	AGCGGTTGTTGATGGGTGAGTC	0.019					
**7697**	C1d1	L07684	TGATCACGCCCTCATAATCATTTTCCTT	0.033	73	*(ct)^23^GTTAGGAAAAGGGCATACAGGA*	0.020	68	C (yellow), T (red)
		H07705	TGTTGTGAGTGTTAGGAAAAGGGCA	0.033					
**14433**	C4c	L14431	GACCTCAACCCCTGACCCCCAT	0.019	57	(ct)^10^ACCCCTGACCCCCATG	0.015	36	C (yellow), T (red)
		H14443	ACTACAGCGATGGCTATTGAGGAG	0.019					
**2092**	D1	L02079	GCCCACAGAACCCTCTAAATCCCC	0.033	75	*(ct)^26^cAGCTGTTCCTCTTTGGACTAACA*	0.020	76	G (blue), A (green)
		H02106	TCCTAGTGTCCAAAGAGCTGTTCCT	0.033					
**9667**	D2a1	L09652	GGAGTATCAATCACCTGAGCTCACCA	0.032	70	*(ct)^24^cCTTGAATTATTTGGTTTCGGTTG*	0.020	72	T (red), C (yellow)
		H09670	GCAGTGCTTGAATTATTTGGTTTCGGT	0.032					
**4991**	D2a2	L04976	TCATAGCAGGCAGTTGAGGTGGA	0.032	88	(ct)^20^GGTGGATTAAACCAAACCCA	0.015	60	G (blue), A (green)
		H05007	TCCTATGTGGGTAATTGAGGAGTATGC	0.032					
**9181**	D2b	L09159	TCGCTGTCGCCTTAATCCAAGCC	0.032	68	*(ct)^12^cTTGTCGTGCAGGTAGAGGC*	0.015	44	T (red), C (yellow)
		H09183	TGTGTTGTCGTGCAGGTAGAGG	0.032					
**10181**	D4b1	L10177	AATCCACCCCTTACGAGTGCGG	0.006	63	*(ct)^13^GGCGGGGGATATAGGGTC*	0.015	44	G (blue), A (green)
		H10197	TTATGGAGAAAGGGACGCGGGC	0.006					
**6285**	D4h3a	L06282	GCCGGAGCAGGAACAGGTTGAA	0.032	75	*(ct)^2^°CCCTGCTAAGGGAGGGTAGA*	0.020	60	C (yellow), T (red)
		H06314	GTCTACGGAGGCTCCAGGGTGG	0.032					

SBE probes in reverse direction are shown in Italics.

hg haplogroup; conc. concentration; bp base pairs; nt nucleotides.

### Ethics statement

All necessary permits were obtained for the described study, which complied with all relevant regulations. Permissions to collect, export and analyze ancient Peruvian specimens from the Huaca Pucllana site were granted by the Ministry of Culture (the former National Institute of Cultural Heritage – INC) and the National Museum of Archaeology, Anthropology and History of Peru (MNAAHP) and are available on request (ACTA No 017-2010-ARMC-MNAAHP-MC and Resolución Viceministerial No. 120-2010-VMPCIC-MC). No specific permits were required for the modern control samples when solely used for methodological validation (waiver from The Human Research Ethics Committee (HREC) at the University of Adelaide). Swab samples were nevertheless collected using written informed consent.

### Samples and DNA extractions

DNA from modern control samples (AC, GV) was extracted from cheek swabs using QIAamp DNA Mini Kit (Qiagen) a following the manufacturer's instructions. Ancient samples were collected by MIBR, BL and WH under DNA-free conditions at the Museo de Sitio Huaca Pucllana, Calle General Borgoño cuadra 8 s/n, Miraflores, Lima, Perú, where the samples are stored. Sample preparation, DNA extractions and PCR amplification from ancient samples were performed at the Australian Centre for Ancient DNA in Adelaide, Australia, applying established methods and authentication criteria as described previously [26], [42], [43]. In brief, we used an in-house silica extraction method, detailed in [26], to extract DNA from two independent samples per individual. PCR amplifications from each extract and direct sequencing of the HVR-I were performed using four overlapping primer pairs with reaction conditions described in [42], [44]. Details of the four primer pairs are given in (Table 2).

**Table 2:** Details of primers used for standard HVR-I amplification and sequencing.

Primer	Primer Sequences 5′ to 3′	Length in bp (incl./excl. primer)	Reference
L16055	GAAGCAGATTTGGGTACCAC	126 (87)	[54]
H16142	ATGTACTACAGGTGGTCAAG		[55]
L16117	TACATTACTGCCAGCCACCAT	162 (115)	[42]
H16233	GCTTTGGAGTTGCAGTTGATGTGT		[42]
L16209	CCCCATGCTTACAAGCAAGT	179 (138)	[54]
H16348	ATGGGGACGAGAAGGGATTTG		[42]
L16287	CACTAGGATACCAACAAACC	162 (122)	[54]
H16410	GCGGGATATTGATTTCACGG		[54]

### Multiplex PCR amplification

PCR amplifications were carried out in a final reaction volume of 12.5 μl consisting of 0.5 μL DNA sample (3 μL for ancient DNA), 1x PCR Gold Buffer, 6.5 mM MgCl_2_, 0.1 U AmpliTaq Gold DNA polymerase (all Applied Biosystems) 1.25 mM dNTP solution (Bioline Pty Ltd), (0.8 μg RSA for ancient DNA samples), and a primer mix consisting of 26 primer pairs, with concentrations given in Table 1. PCR was carried out on a Tetrad 2 Peltier Thermal Cycler (Bio-Rad Laboratories) using the following conditions: 95°C for 6 min and 30 cycles (45 cycles for ancient DNA samples) of 95°C for 30 s, 55°C for 30 s, 65°C for 30 s, and a final extension time at 65°C for 6 min. Amplification success was monitored via gel electrophoresis on an 3.5% agarose gel (100 V for 40 min; Hyperladder V DNA size ladder (Bioline Pty Ltd)). PCR products were purified by mixing 5 μl of PCR reaction with 1 U ExoSAP-IT (Thermo Fisher Scientific Australia Pty Ltd), followed by incubation at 37°C for 50 min, 80°C for 15 min and 15°C for 10 min. Single Base Extension reactions consisted of a final volume of 5 μL containing 1 μL PCR product, 2.5 μL SNaPshot ready reaction mix (Applied Biosystems), and 0.5 μL extension primer mix (individual concentrations are given in Table 1). Thermocycling of the SBE reactions was performed in a Tetrad 2 Peltier Thermal Cycler (Bio-Rad Laboratories) with the following conditions: 96°C for 10 s; followed by 35 cycles of 55°C for 5 s and 60°C for 30 s. SBE products were purified by adding 1 U Shrimp Alkaline Phosphatase (Thermo Fisher Scientific) to the reaction solution and incubating it at 37°C for 50 min, 80°C for 15 min and 15°C for 10 min.

Capillary electrophoresis was performed on a 3130 xl Genetic Analyser (Applied Biosystems) using POP-6 polymer and a customised run module, by adding 1 μL sample DNA to 18.5 μL Hi-Di Formamide and 0.5 μL GeneScan-120 LIZ internal size standard (Applied Biosystems). Electropherograms were analysed using the software Genemapper ID version 3.2.1 software (Applied Biosystems) applying custom panel and bin settings available on request.

### Sensitivity tests

We performed sensitivity studies using serial dilutions of 1, 1∶10, 1∶100, 1∶1000 and 1∶10,000 of DNA from a buccal swab sample from a lab member (AC). The mtDNA copy number of the modern sample was determined through qPCR using the SYBR-Green kit (Qiagen), targeting a short 77 bp fragment of human mitochondrial DNA with primer pair L13258 and H13295 [45]. Serial dilutions were treated as separate samples, and each sample was analysed in triplicate. The qPCR reaction was performed in a total reaction volume of 10 μL consisting of 1 μL of each sample dilution, 2x Brilliant SYBR Green Master Mix and 0.1 μM of each primer. The qPCR were carried out on a Rotor-Gene Q Real-Time PCR cycler (Qiagen) with thermocycling conditions as follows: 95°C for 5 min, followed by 45 cycles of 95°C for 10 s, 58°C for 20 s and 72°C for 20 s.

## Results and Discussion

### Optimization of the multiplex protocol

The AmericaPlex26 assay was initially tested with default concentrations of 0.017 μM for each primer (3 μL of 25 μM stock) and 0.015 μM for each SBE primer (3 μL of 50 μM stock) to assess the generic efficiency of primers or probes when used in the multiplex assay. Twenty-two out of 26 SNP sites could be readily amplified, albeit with highly variable peak heights across the assay. Primers and probes for the four problematic SNP sites were each tested in singleplex PCR and SNaPshot reactions to ensure they performed individually as expected. If the SNP fragment were successfully amplified in the singleplex PCR, concentrations of the primer would be doubled in the following multiplex PCR reaction mix.

We chose 3000 relative fluorescence units (rfu) as a default average peak height based on the ancestral allele status observed in our European modern control sample (AC), and calculated the percentage difference between peaks and the 3000 rfu average. Multiplex primer concentrations were adjusted according to this percentage difference to allow amplification of problematic SNP sites and to balance the peak heights of those that did amplify. Based on poor performance of the primer pair chosen to amplify the C1b SNP site (A493G), we performed a second round of balancing primer concentration with a new primer pair for this site.

**Figure 2:**
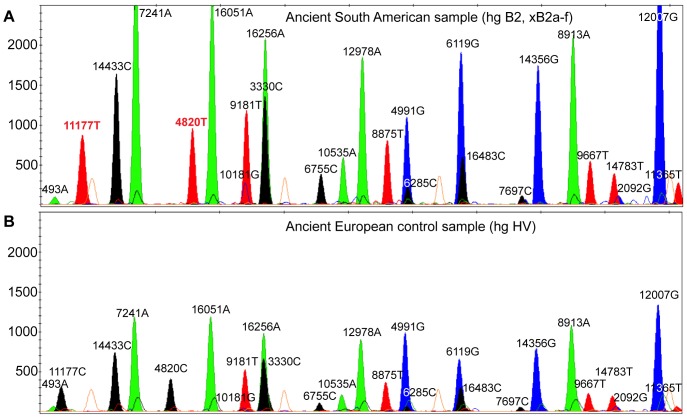
Electropherograms of two ancient examples representing the optimized AmericaPlex26. Panel A shows a South American sample and panel B an ancient European sample illustrating the ancestral state of all 26-haplogroups B2a (16483), B2b (6755), B2c (7241), B2d (8875), B2e (6119) and B2f (10535).

To further refine the balance in peak height, the concentration of some SBE extension primers was adjusted to the final recommended concentrations given in Table 1. Changes to probe concentrations resulted in a more balanced electropherogram and amplification of all 26 SNP sites using modern buccal swab and ancient DNA samples (Figure 2).

### Sensitivity studies

**Figure 3:**
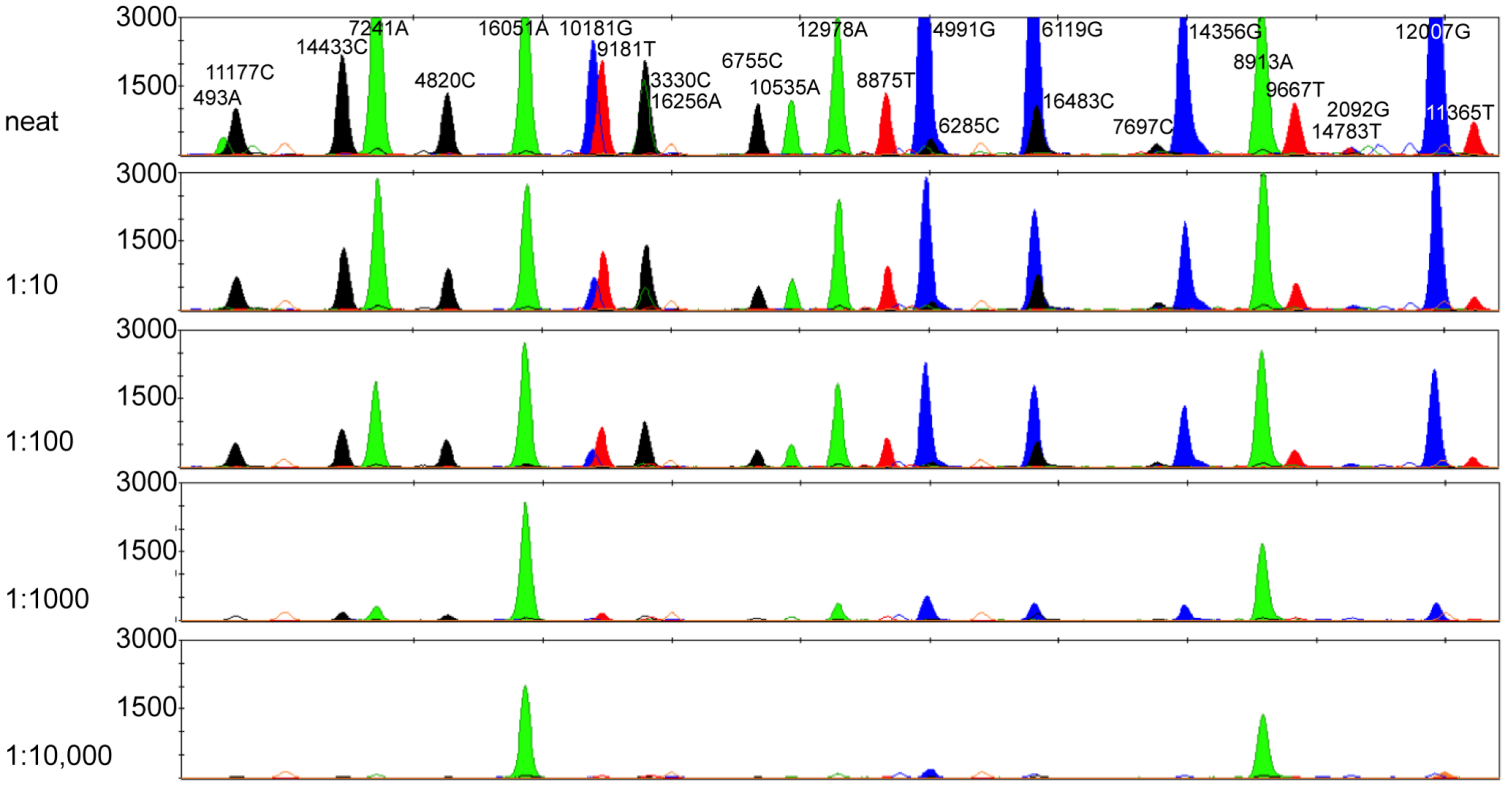
Electropherograms from the sensitivity tests showing four serial dilutions of template DNA with a starting copy number of 1,171,699 copies/μL. Note the increasing number of locus dropout as template DNA concentration decreases.

The amount of mitochondrial DNA was measured for a modern sample (1,171,699 copies/μL) and four serial dilutions of 1∶10, 1∶100, 1∶1000 and 1∶10,000 using real-time quantitative PCR (Figure 3). A near complete SNP profile could be observed for serial dilutions up to 28,278 copies/μL DNA (1∶100), which is similar to other published multiplex assays [23], [24], [46].

### Method application on ancient samples

**Figure 4:**
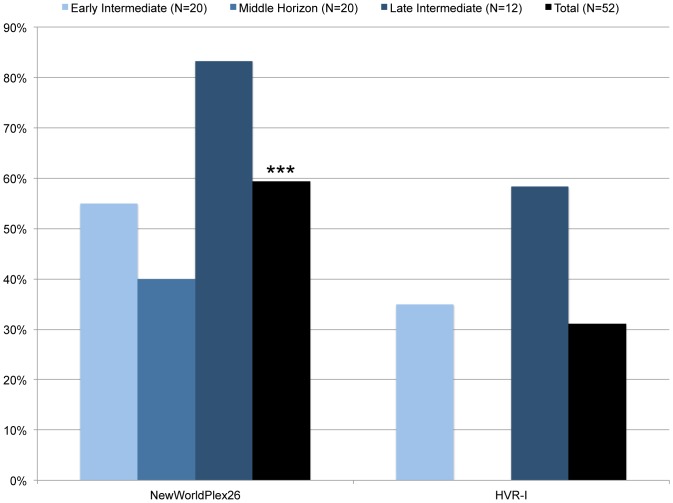
Genotyping success of the AmericaPlex26 assay compared to standard HVR-I sequencing via four overlapping amplicons. The success rate is given in percentage of unambiguous genotype calls for each of the two methods per cultural horizon and all results combined (black bars, p<0.0001).

The AmericaPlex26 assay was tested on ancient samples from three successive pre-Columbian cultures from the Huaca Pucllana archaeological site in Lima, Peru. They included samples from the Early Intermediate (n = 20; 200–600 AD), the Middle Horizon (n = 20; 600–1000 AD) and the Late Intermediate (n = 12; 1000–1476 AD) [47] plus an Early Medieval European samples as control for the ancestral state. Samples from each period varied in the state of preservation, due to differences in mortuary customs. From our test dataset of 52 samples in total, we were able to unambiguously type 29 samples (56%) (Table 3, Figure 4). A typing result was considered reliable when two samples from the same individual could be unambiguously assigned to the same sub-hg in two independent experiments.

**Table 3:** Direct comparison of results for HVR-I sequencing and AmericaPlex26 SNP typing assay for samples unambiguously typed using the AmericaPlex26 assay.

Individual	Museum no.	Samples	HVR I hg	AmericaPlex26	Consensus
EI 1	A06 95/96	**10802A**	?	B2	B2
		**10803A**	?	B2	
EI 2	A06 79/01 Ind2	**10804A**	D1	D1?	D1
		**10805A**	D1	M	
EI 3	A06 76/96	**10809A**	C1	C1b	C1b
		**10810A**	C1	C1b	
EI 4	A15 06/00	**10813A**	B4	B2	B2
		**10814A**	B4	B2	
EI 5	A06 01/02	**10817A**	C1	C1b	C1b
		**10818A**	?	C1b	
EI 6	A6 68/96	**10821A**	B4	B2	B2
		**10822A**	B4	B2	
EI7	A06 90 Ind1	**10787A**	?	B2	B2
		**10788A**	?	B2	
EI 8	A20 05/09	**10789A**	B4	B2b	B2b
		**10790A**	?	B2b	
EI 9	A20 03/07 Ind1	**10791A**	A2	A2	A2
		**10792A**	A2	A2	
EI 10	A06 82/96	**10793A**	B4	B2	B2
		**10794A**	B4	B2	
EI 11	A06 77/96	**10796A**	B4	B2b	B2
		**10797A**	?	B2	
EI 12	A06 79/96 Ind1	**10800A**	B4	B2b	B2
		**10801A**	B4	B2	
MH 1	A20 08/08 Ind2	**10733A**	?	C1b	C1b
		**10734A**	?	C1b	
MH 2	A20 CF003/09	**10741A**	?	B2	B2
		**10742A**	?	B2	
MH 3	A20 07/08 Ind4	**10749A**	?	B2	B2
		**10750A**	?	B2	
MH 4	A20 05/08	**10753A**	?	B2	B2
		**10754A**	B4?	B2	
MH 5	A20 18/08	**10765A**	-	C1b	C1b
		**10766A**	-	C1b	
MH 6	A20 01/09 Ind1	**10772A**	C1	C1b	C1b
		**10773A**	?	C1b	
MH 7	A20 01/09 Ind2	**10774A**	?	A2	A2
		**10775A**	-	A2	
MH 8	A20 04/07 Ind2	**10778A**	D?	A2	A2
		**10779A**	-	A2	
LI 1	A15 01/02	**10709A**	C1	M	C1
		**10710A**	C1	C1b	
LI 2	A0 cf14 ind-1/98	**10712A**	B4	B2	B2
		**10713A**	B4	B2	
LI 3	A01 CF16/98	**10715A**	?	B2	B2
		**10716A**	-	B2	
LI 4	A0 CF15/01	**10717A**	C1	M	M
		**10718A**	?	M	
LI 5	A15 CF36/01	**10719A**	B4	B2	B2
		**10720A**	B4	B2b	
LI 6	A15 Sin Contexto	**10724A**	C1	C1b	C1b
		**10725A**	C1	C1b	
LI 7	A3 CF01/04	**10726A**	?	B2b	B2
		**10727A**	?	B2	
LI 8	A0 08/98	**10728A**	B4	B2	B2
		**10729A**	B4	B2	
LI 9	A15 02/02	**10730A**	B4	B2	B2
		**10730Y**	B4	B2b	
LI 10	A0 56/97	**10731A**	C1	C1b	C1b
		**10731Y**	C1	C1b	

Consensus haplogroups were called based on last common SNP from both replicates from independent extractions, and minimum peak size >50 rfu. (?)/(−): Insufficient or no sequence information; EI: Early Intermediate; MH: Middle Horizon; LI: Late Intermediate.

We subsequently compared the AmericaPlex26 assay results to our previous attempts at amplifying and sequencing the mitochondrial HVR-I with four overlapping primer pairs and found that the AmericaPlex26 assay improved the typing efficiency from ancient samples (Table 3, Figure 4). For example, the AmericaPlex26 assay allowed reliable SNP typing for eleven Early Intermediate (55%) and ten Late Intermediate samples (83%), whereas HVR-I sequencing gave reliable sequence haplotypes for seven (35%) and seven (58%) samples, respectively. For example, HVR-I sequencing for samples 10802A and 10803A failed, while the AmericaPlex26 assay revealed specific hg B2 (Table 3). Samples from the Middle Horizon culture were in general less well preserved, resulting in eight consensus sub-hg calls (40%) using the AmericaPlex26 assay, whereas HVR-I sequencing did not produce any reliable sequence haplotype from the sample replicates (0%). This highlights the genotyping power of our assay when dealing with challenging samples.

Importantly, SNP typing with the AmericaPlex26 assay also gave a higher resolution compared to traditional HVR-I sequencing. For example, samples 10809A and 10810A of the Early Intermediate period were assigned to the major hg C1 by HVR-I sequencing, yet the AmericaPlex26 assay allowed further resolution to sub-hg C1b. In addition, while many of the HVR-I results from Late Intermediate samples remained tentative, i.e. non-reproducible, the AmericaPlex26 assay provided reliable and specific sub-hgs for both replicates (Table 3, Figure 4). Taking all results together, the AmericaPlex26 assay showed a significantly higher success rate when compared to the standard HVR-I sequencing (p<0.0001, Wilcoxon matched-pairs signed rank test; Figure 4). This is likely due to the difference in amplicon sizes between the two methods.

### The overall effectiveness of the assay

Overall, the effectiveness of the multiplex SNaPshot method in analysing ancient DNA lies in the fact that it only requires minimal flanking regions either side of the SNP, which in theory allows the design of very short overall amplicon sizes [40], [48], 56–90 bp in our case. This makes it suitable for the extremely fragmented and damaged state of ancient DNA, while the multiplex approach maximises the amount of information that can be gained per PCR [15], [49], [50]. As such, SNaPshot typing is able to generate results for samples for which traditional sequencing methods often fail with ancient and/or degraded DNA, as they require longer fragment lengths to be cost-effective [51]. Multiplexing also allows the combination of many informative SNP sites into one reaction, which are otherwise spread across longer sequence regions. On its own, the multiplex PCR and SNaPshot method is time- and cost-effective, and requires substantially smaller amounts of valuable DNA extract compared to HVR-I sequencing, as fewer individual reactions are needed from preparation of the multiplex PCR to capillary electrophoresis [48], [50].

We show that the AmericaPlex26 can be used to complement or expand upon standard mtDNA sequencing approaches for ancient Native American populations, and especially for ancient samples where DNA preservation does not allow amplification of longer (>100 bp or more) DNA molecules. It efficiently and economically targets characteristic SNPs from the coding region of mtDNA [15] in order to corroborate HVR-I sequencing results and to define a particular sub-hg [14]. Alternatively, the AmericaPlex26 can be used in addition to global mtDNA SNP multiplexes, such as the GenoCore22 and others [23], [24]. Moreover, it is flexible enough to add newly discovered SNPs/lineages in order to enhance sub-regional resolution (see e.g. [32], [35].

In our experience, the new method provided an extremely useful one-reaction test to screen larger numbers of degraded samples allowing the assessment of the general state of preservation, the authenticity of the result (i.e. absence of potential contaminating lineages), while at the same time allowing a categorisation of potentially interesting sub-hgs. We are currently using this approach in order to further dissect the phylogenetic resolution via DNA library creation and targeted mtDNA enrichment and Next Generation Sequencing [25], [26].

## Conclusions

We present a powerful, optimized SNP assay, which allows unambiguous typing of Native American mtDNA ‘founder lineages’ and additional SNPs for further resolution. This short-amplicon AmericaPlex26 assay is highly efficient, time and cost-effective compared to classical HVR-I sequencing, and allows highly resolved SNP typing of degraded DNA samples in forensic and ancient DNA work. It is suitable as a qualitative ‘screening’ method to identify samples with sufficient DNA preservation, free of contaminants that complicate full mitochondrial sequencing (and beyond) via Next Generation Sequencing techniques.
